# A Benign Rectal Polyp With Osseous Metaplasia: A Pediatric Case Report and Literature Review

**DOI:** 10.7759/cureus.33052

**Published:** 2022-12-28

**Authors:** Tuqa A Alsinan, Mohammad Almohaidly, Mohammad AlOnazi, Ahmed Beltagi, Salman A Alfaifi

**Affiliations:** 1 Department of Pediatric Surgery, Prince Sultan Military Medical City, Riyadh, SAU; 2 Graduate Medical Education, Alfaisal University College of Medicine, Riyadh, SAU; 3 Department of Histopathology, Prince Sultan Military Medical City, Riyadh, SAU

**Keywords:** benign colorectal polyp, histopathology, pediatric surgery, osseous metaplasia, rectal polyp

## Abstract

Osseous metaplasia is rarely detected in benign and malignant lesions in the gastrointestinal tract, especially in the pediatric population under the age of six. Here, we report the case of a four-year-old boy with a history of constipation associated with painless bleeding per rectum for six months which was excised successfully. Microscopically, there was osseous metaplasia with no malignant changes. Further reporting is needed due to the limited cases of osseous metaplasia in colorectal polyps, with unknown mechanisms of pathogenesis and prognostic factors, specifically in the pediatric population.

## Introduction

Benign gastrointestinal polyps are the most common type of polyps in children. They are benign masses of normal tissue that may occur sporadically or are inheritable with other syndromes [1,2]. Osseous metaplasia is rarely detected in benign and malignant lesions in the gastrointestinal tract, especially in the pediatric population under the age of six. Metaplasia is defined as the replacement of a typical differentiated cell type with another one that presents outside its tissue [1]. Although more than 20 previous cases have reported osseous metaplasia, only seven are in inflammatory rectal polyps [3-5]. The exact pathogenesis is not fully understood; however, several mechanisms and theories of the chronic inflammatory process and metaplastic changes have been investigated [1-3]. Usually, detecting such histopathological findings does not require further investigation or a specific treatment plan [2,3]. This case reports a benign rectal polyp with ulceration and osseous metaplasia without malignant changes in a four-year-old boy.

## Case presentation

**Figure 1:**
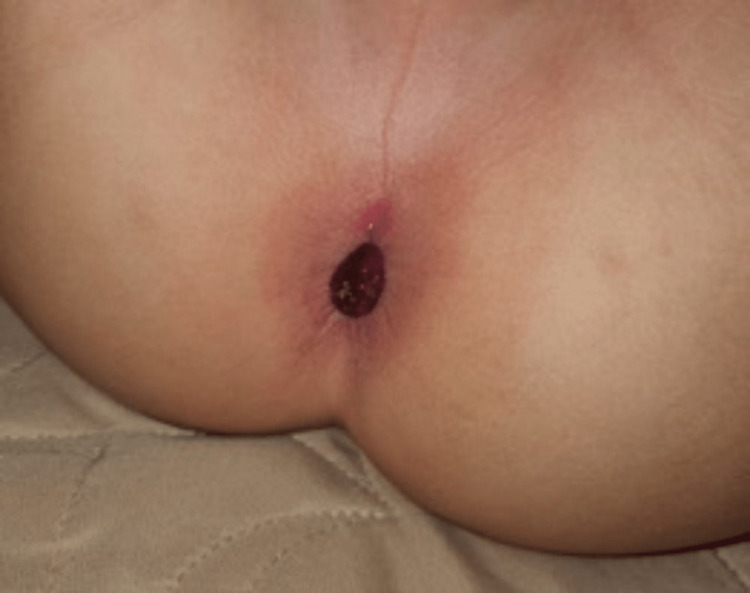
Protruding anal mass.

A four-year-old boy presented to the Emergency Department (ED) with a history of constipation for two months, followed by painless bleeding per rectum with a protruding anal mass (Figure 1).

He was diagnosed with an anal fissure and managed conservatively with pediatric gastroenterology with no improvement. There was no history of fever, rash, joint pain, vomiting, or weight loss. There was a family history of celiac disease with no other significant chronic illnesses. He was referred to a pediatric surgery clinic for further management. Upon physical examination, the patient was afebrile and vitals were within normal limits. A digital rectal examination revealed a pedunculated posterior rectal polyp at 6 o’clock. The remainder of the physical examination was unremarkable. Laboratory tests were all within the normal range. The parents were counseled for surgical polypectomy under general anesthesia.

**Figure 2:**
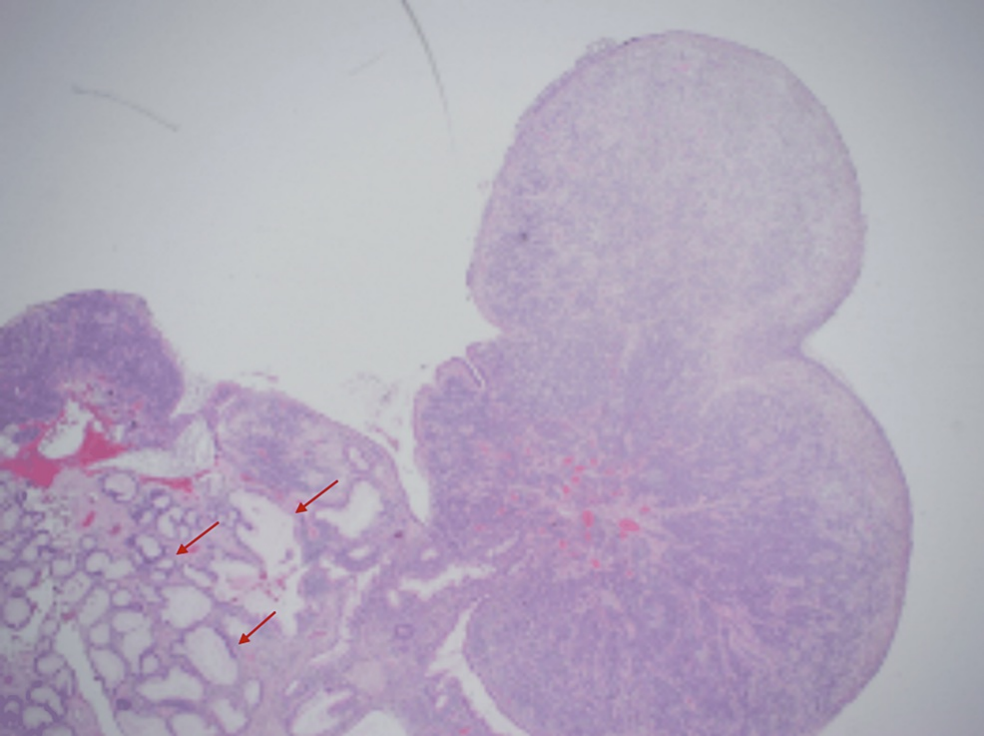
Polypoid tissue with surface ulceration and cystic dilated glands (red arrows).

**Figure 3:**
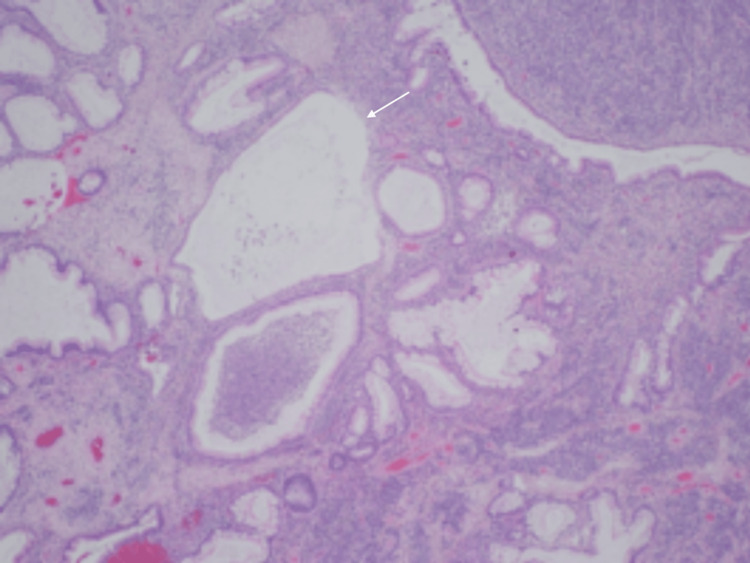
Dilated glands filled with mucus and inflammatory debris (white arrow).

**Figure 4:**
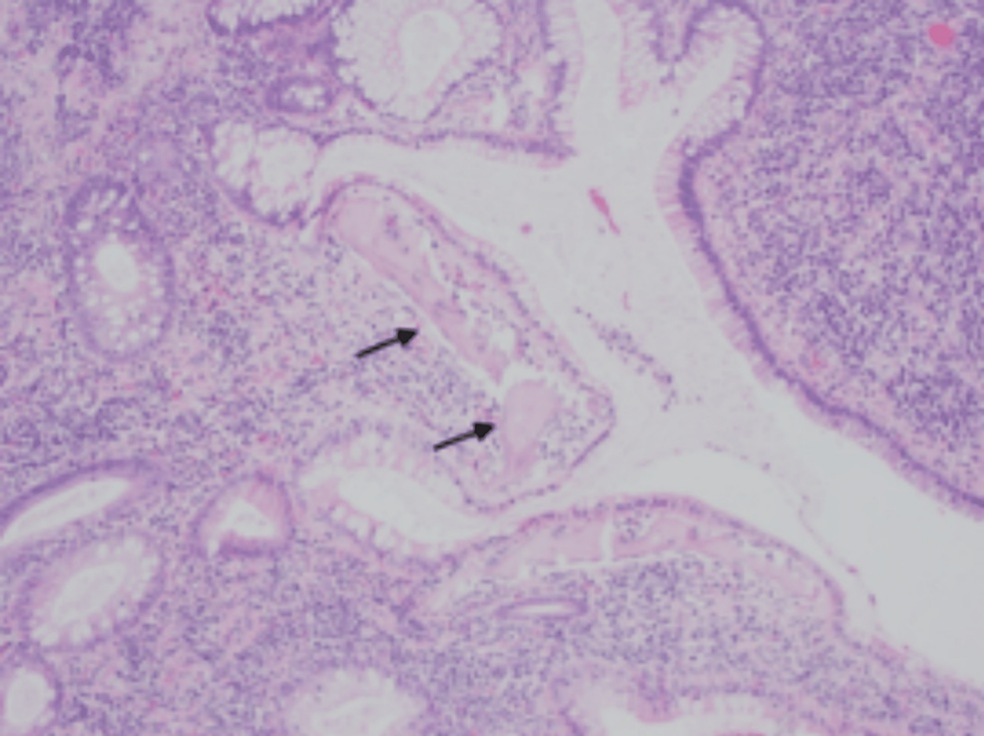
Cystically dilated glands with osseous metaplasia showing the bony trabeculae (black arrows).

The polyp was then excised successfully, with no other polypoid lesions detected. The polyp was located around 8-10 cm above the dentate line and was carefully excised. On gross examination, a white-tan, soft, polypoidal tissue measuring 1.0 × 0.8 × 0.5 cm and 0.4 × 0.3 × 0.3 cm was noted. Microscopically, a hamartomatous polyp with ulcerations and osseous metaplasia was noted. The tissue showed histological changes that were discussed with the pathologist (Figures 2-4).

The patient tolerated the procedure well, with no immediate or delayed complications. He was discharged with instructions, medical management for constipation, and regular follow-up.

## Discussion

Osseous metaplasia is a well-known phenomenon found in various organs, such as the nasopharynx and endocervix; however, the rectal polyp is rarely reported, with limited previously reported cases in the literature [5]. Benign polyps are the most common type of polyps in children. Metaplasia is defined as the replacement of a typical differentiated cell type with another one that presents outside its tissue or as a reprogramming of the undifferentiated stem cells in response to stress leading to chronic inflammatory changes [1,2]. Around 21 previous cases have reported osseous metaplasia. The patients present clinically with a history of painless rectal bleeding with or without prolapsed rectal mass [1-6]. Further, the age variation of osseous metaplasia cases is between nine and 39 years, with a few elderly cases above 70 years old [5]. Moreover, the size of rectal polyps varies between 8 and 12 mm, with a male predominance [1-7]. Although the exact pathogenesis is not fully understood, it is mainly associated with a chronic inflammatory response to the muscularis mucosa, mucosal prolapse, and chronic stress or trauma during defecation and intestinal movements and can be related to some inheritable conditions such as juvenile polyposis syndrome [2,6]. Usually, detecting such histopathological findings with expected cystic changes in the dilated glands’ epithelium from cuboidal to columnar with some inflammatory cells does not require further investigation or a specific treatment plan [2,3].

Interestingly, almost all pediatric cases are inflammatory polyps with an equal male-to-female ratio [1-6]. The clinical features and prognostic factors remain unknown [2]. Very few cases have been reported in the past five years, with only one case reported in Saudi Arabia in 2019 of a six-year-old boy [1-6]. There are limitations in the literature in reporting and discussing the outcomes of osseous metaplasia in pediatric benign rectal polyps, warranting further investigation.

Table 1 summarizes the inflammatory rectal polyps histologically associated with osseous metaplasia in the pediatric population over the past seven years.

**Table 1:** A literature review of rectal polyps with osseous metaplasia in the pediatric population over the past seven years.

Year	Country	Age	Gender	Type of polyp	Location	Pathology
2022	Saudi Arabia (current case)	4-year-old	M	Inflammatory	Rectum	Benign
2021	Turkey [1]	7-year-old	F	Inflammatory	Rectum	Benign
2020	United States [5]	17-year-old	M	Inflammatory	Rectum	Benign
2020	Iran [2]	10-year-old	F	Inflammatory	Rectum	Benign
2020	Iran [2]	4-year-old	M	Inflammatory	Rectum	Benign
2020	India [6]	9-year-old	F	NA	Rectum	Hyperplastic
2018	Saudi Arabia [4]	10-year-old	M	Inflammatory	Rectum	Benign
2018	United Kingdom [3]	6-year-old	M	Inflammatory	Rectosigmoid	NA
2018	NA [7]	8-year-old	M	Inflammatory	Rectum	Benign
2018	NA [7]	2-year-old	F	Inflammatory	Rectum	Benign
2018	NA [7]	4-year-old	M	Inflammatory	Rectum	Benign

## Conclusions

We reported a rare and incidental histopathological finding of osseous metaplasia in a young boy with a prolapsed rectal polyp associated with painless bleeding. Cases of osseous metaplasia in colorectal polyps are few, with unknown mechanisms of pathogenesis and prognostic factors, specifically in the pediatric population. Benign osseous metaplasia should be considered in all colorectal lesions as a differential diagnosis; however, malignant lesions must be investigated and ruled out. We believe this is the second case reported in the literature in Saudi Arabia, and only 21 cases have been reported worldwide. Reporting such findings and understanding the underlying mechanisms would help in determining its outcomes.
